# Induction of Endoplasmic Reticulum Stress Response by the Indole-3-Carbinol Cyclic Tetrameric Derivative CTet in Human Breast Cancer Cell Lines

**DOI:** 10.1371/journal.pone.0043249

**Published:** 2012-08-14

**Authors:** Luca Galluzzi, Mauro De Santi, Rita Crinelli, Cinzia De Marco, Nadia Zaffaroni, Andrea Duranti, Giorgio Brandi, Mauro Magnani

**Affiliations:** 1 Department of Biomolecular Science, University of Urbino “Carlo Bo", Fano (PU), Italy; 2 Department of Biomolecular Science, University of Urbino “Carlo Bo", Urbino (PU), Italy; 3 Department of Experimental Oncology and Molecular Medicine, Fondazione IRCCS Istituto Nazionale dei Tumori, Milano, Italy; 4 Department of Biomolecular Science, Medicinal Chemistry and Technology Unit, University of Urbino “Carlo Bo", Urbino (PU), Italy; Wayne State University School of Medicine, United States of America

## Abstract

**Background:**

Indole-3-carbinol and its metabolic products are considered promising chemopreventive and anticancer agents. Previously we have shown that the indole-3-carbinol cyclic tetrameric derivative CTet induces autophagy and inhibits cell proliferation via inhibition of Akt activity and overexpression of p21/CDKN1A and GADD45A, in both estrogen receptor-positive (MCF-7) and triple negative (MDA-MB-231) breast cancer cell lines. In the present study, we further characterize the autophagic response and investigate the mechanism through which CTet regulates these events.

**Methodology/Principal Findings:**

Analysis of gene expression microarray data and subsequent confirmation by quantitative real-time PCR, showed that CTet is able to induce up-regulation of key signaling molecules involved in endoplasmic reticulum (ER) stress response (e.g. DDIT3/CHOP, CHAC1, ATF3, HSPA5/BiP/GRP78, CEBPB, ASNS) and autophagy (e.g. MAP1LC3B), in both MCF-7 and MDA-MB-231 cell lines. Moreover, the monitoring of Xbp-1 splicing confirmed the activation of IRE1/Xbp-1 ER stress response branch after CTet treatment. The role of autophagic processes (known to be induced by ER stress) was investigated further through ATG5 gene silencing and pharmacological inhibition of AVOs formation. CTet was shown to induce an autophagy-related cell death. Moreover, CTet-treated cells stained with Hoechst/PI revealed the presence of necrotic processes without evidence of apoptosis.

**Conclusions/Significance:**

The ER stress response was identified as the main upstream molecular mechanism through which CTet acts in both hormone-responsive and triple-negative breast cancer cells. Because of its important role in cancer development, ER stress is a potential target in cancer therapy. The abiltiy of CTet to induce ER stress response and subsequently activate a death program in tumor cells confirms this molecule as a promising anticancer agent.

## Introduction

Indole-3-carbinol (I3C) and its derivatives exhibit antitumor activity in various cancer cell lines. The I3C derivatives characterized by antiproliferative properties, such as DIM (3,3′-diindoylmethane) and CTet (a cyclic tetramer), have been shown to induce a wide spectrum of signaling targets and cellular responses such as apoptosis and/or cell cycle arrest [1].

We have recently shown that CTet induces autophagy and inhibits cell proliferation in both estrogen receptor-positive (MCF-7) and triple negative (MDA-MB-231) breast cancer cell lines. Moreover, the inhibition of Akt activity and p53-independent p21/CDKN1A and GADD45A overexpression have been identified as the main molecular events responsible for CTet activity in both cell lines [2]. However, the mechanism through which CTet regulates these events remains unclear.

Autophagy, a conserved cellular pathway activated in response to starvation and after treatment with some chemotherapeutic drugs, can also be induced by endoplasmic reticulum (ER) stress [3]. The ER stress is caused by perturbation of ER functions (i.e. protein synthesis/folding/post-translational modifications, biosynthesis of lipids and sterols, Ca^2+^ storage), and it is sensed by three ER-transmembrane transducers: ATF6, IRE1 and PERK [4], [5]. IRE1 and PERK are activated by phosphorylation, while ATF6 is translocated to the Golgi apparatus and cleaved by intramembrane proteolysis to release the transcriptionally active N-terminal domain. The activated ATF6 stimulates the expression of genes containing ER stress elements (ERSE-I, -II), UPR elements (UPRE), and cAMP response elements (CRE) in their promoters [4]. The activated IRE1 induces the unconventional splicing of X-box binding protein 1 (Xbp-1) mRNA [6]. In metazoans, a 26-nucleotide intron is spliced out, leading to a spliced form of Xbp-1 mRNA (sXbp-1) which encodes a highly active transcription factor belonging to the basic-leucine zipper (bZIP) family. The sXbp-1 protein induces the expression of several genes encoding ER chaperones (e.g. HSPA5/BiP/GRP78, ERdj4/DNAJB9) [7] and proteins involved in ER-associated protein degradation (ERAD) (e.g. HERPUD1, HRD1) [8], [9]. PERK (EIF2AK3) is a protein kinase which phosphorylates the alpha subunit of eukaryotic initiation factor 2 (eIF2α), leading to global translation attenuation. At the same time, phosphorylated eIF2α induces selective translation of activating transcription factor 4 (ATF4). ATF4, in turn, induces the expression of several genes including amino acid transporters, chaperones, and C/EBP homologous protein (DDIT3/CHOP). Together, these three branches mitigate ER stress by reducing protein synthesis, facilitating protein degradation, and increasing production of chaperones. One consequence of ER stress is the accumulation of reactive oxygen species (ROS) that promotes a state of oxidative stress. PERK signaling also engages survival responses against oxidative stress by inducing the expression of genes involved in the oxidative stress response [10]. When ER stress is prolonged and it is not possible to recover the ER function, the apoptotic pathway is activated [5]. To repair tissue damage caused by cell death, the ER stress also induces an inflammatory response through the expression of several inflammatory cytokines (e.g. IL-6, IL-8) [11].

The ER stress has an important role in cancer development, therefore being a potential target in cancer therapy [12]. The artificial induction of ER stress response in tumor cells, which causes the activation of a death program, may be used in the development of anticancer drugs. Several anticancer compounds eliciting ER stress response have been described by Healy et al. [12].

This study was aimed to investigate the specific stress response pathways activated by CTet in estrogen receptor-positive (MCF-7) and triple negative (MDA-MB-231) breast cancer cell lines, by looking for up-regulation/activation of key signaling molecules, and further characterization of the autophagic response. Induction of ER stress response, together with autophagy-related cell death were identified as major consequences of CTet treatment of the breast cancer cells tested.

## Results

### Genes Induced by CTet in MCF-7 and MDA-MB-231 Cells are Related to ER Stress Response

Microarray analysis has been used to examine the transcriptional response elicited by CTet treatment in MCF-7 and MDA-MB-231 cell lines [2]. Complete microarray data are available in ArrayExpress database (accession number: E-MEXP-2989). In the present investigation a list of 116 genes significantly up-regulated in both cell lines after 24 h treatment with 6 µM and 12 µM CTet [2] was pruned by hand to delete duplicate genes or genes with no associated ontologies, obtaining a list of 92 up-regulated genes with known functions. Among these genes, a relatively large number of genes with roles in ER stress response and related functions, such as response to starvation and autophagy were identified by searching literature (Table 1).

**Table 1:** Partial list of genes related to ER stress and autophagy/starvation that are up-regulated in CTet-treated MCF-7 and MDA-MB-231 cells.

			Fold change					
Accession N. Gene Title		Gene ID	MCF-7			MDA-MB-231		
*ER stress-related transcripts*			CTet 6µM	CTet 12µM		CTet 6µM		CTet 12µM
NM_004024	activating transcription factor 3	ATF3	11.58	30.40		2.45		5.36
NM_133436	asparagine synthetase, transcript variant 1	ASNS	1.85	2.44		1.69		2.66
NM_005980	S100 calcium binding protein P	S100P	1.20	2.12		2.11		4.27
NM_019058	DNA-damage-inducible transcript 4	DDIT4	4.91	7.13		3.89		5.71
S62138	TLS/CHOP = hybrid gene (translocation breakpoint)	FUS	3.98	8.84		1.84		2.45
NM_024111	ChaC, cation transport regulator homolog 1 (E. coli)	CHAC1	4.35	4.86		2.78		3.76
NM_021158	tribbles homolog 3 (Drosophila)	TRIB3	1.28	2.07		1.88		2.91
NM_012328	DnaJ (Hsp40) homolog, subfamily B, member 9	DNAJB9	2.67	5.83		2.03		3.32
NM_007034	DnaJ (Hsp40) homolog, subfamily B, member 4	DNAJB4	2.78	4.56		1.93		2.01
NM_006145	DnaJ (Hsp40) homolog, subfamily B, member 1	DNAJB1	1.27	3.08		1.94		2.38
NM_018602	DnaJ (Hsp40) homolog, subfamily A, member 4	DNAJA4	1.30	2.54		1.55		1.85
NM_005346	heat shock 70kDa protein 1B	HSPA1B	1.42	2.61		3.99		4.87
NM_005345	heat shock 70kDa protein 1A	HSPA1A	1.42	2.49		6.18		6.78
NM_019891	ERO1-like beta (S cerevisiae)	ERO1LB	1.97	4.41		2.31		3.43
NM_004235	Kruppel-like factor 4 (gut)	KLF4	1.41	2.69		1.34		1.93
NM_172230	HRD1 protein (HRD1), transcript variant 2	SYVN1	1.56	2.42		1.05		1.69
NM_203418	regulator of calcineurin 1	RCAN1	2.68	4.79		2.02		3.16
NM_000584	interleukin 8	IL8	26.93	85.72		3.59		3.34
NM_000600	interleukin 6 (interferon, beta 2)	IL6	3.68	12.32		4.92		9.98
NM_000963	prostaglandin-endoperoxide synthase 2	PTGS2	1.00	2.49		20.28		36.65
NM_006096	N-myc downstream regulated gene 1	NDRG1	1.65	2.19		1.30		2.02
NM_005194	CCAAT/enhancer binding protein (C/EBP), beta	CEBPB	2.60	5.04		1.63		2.08
NM_000389	cyclin-dependent kinase inhibitor 1A (p21, Cip1)	CDKN1A	1.41	2.27		1.72		2.09
NM_000636	superoxide dismutase 2, mitochondrial	SOD2	1.43	3.13		1.90		2.77
NM_002133	heme oxygenase (decycling) 1	HMOX1	8.09	15.79		2.75		3.21
NM_182743	thioredoxin reductase 1, transcript variant 4	TXNRD1	3.33	5.34		2.06		2.43
NM_014417	BCL2 binding component 3	BBC3	2.31	4.35		2.87		3.79
*Autophagy/starvation-related transcripts*								
BU942678	sequestosome 1	SQSTM1	1.48		2.31		2.00	2.15
NM_003806	harakiri, BCL2 interacting protein (contains only BH3 domain)	HRK	2.05		4.44		1.28	2.83
NM_017983	WD repeat domain, phosphoinositide interacting 1	WIPI1	2.19		4.76		1.58	2.06
NM_021127	phorbol-12-myristate-13-acetate-induced protein 1	PMAIP1	3.56		7.18		1.47	1.86
NM_133436	asparagine synthetase, transcript variant 1	ASNS	1.85		2.44		1.69	2.66
NM_031412	GABA(A) receptor-associated protein like 1	GABARAPL1	3.17		7.30		1.68	2.58
NM_022818	microtubule-associated protein 1 light chain 3 beta	MAP1LC3B	2.05		3.77		1.29	1.62
NM_004281	BCL2-associated athanogene 3	BAG3	1.17		2.32		1.51	1.66

Furthermore, DNA microarray data were also re-analyzed using GeneSifter software (www.genesifter.net; Geospiza Inc., Seattle, WA) with less stringent statistical and/or fold change threshold parameters to identify other target genes considered as ER stress markers but not present in the list of 92 up-regulated genes. This analysis allowed us to identify 4 more up-regulated ER stress marker genes: PERK/EIF2AK3, HERPUD1/HERP (fold change threshold = 1.5; ANOVA, Benjamini–Hochberg false discovery rate correction, *p*<0.01); PPP1R15A/GADD34 (fold change threshold = 1.8; ANOVA, Benjamini–Hochberg false discovery rate correction, *p*<0.05); HSPA5/BiP/GRP78 (fold change threshold = 2; ANOVA, *p*<0.05).

### Real Time-quantitative PCR (RT-qPCR) Analysis of Selected ER Stress Response Genes

The microarray data have been validated on selected targets by RT-qPCR and immunoblot analysis [2]. In this study, we used RT-qPCR to investigate the changes in gene expression of 7 selected genes associated with ER stress and autophagy: DDIT3/CHOP, CHAC1, ATF3, HSPA5/BiP/GRP78, CEBPB, ASNS and MAP1LC3B. All the genes except for HSPA5/BiP/GRP78 were in the list of 92 up-regulated genes. The HSPA5/BiP/GRP78 gene was also chosen for RT-qPCR assay because it was up-regulated with the lowest significance compared to all the ER stress markers identified by microarray analysis, and because it is a major ER chaperone generally induced by ER stress [13].

**Figure 1:**
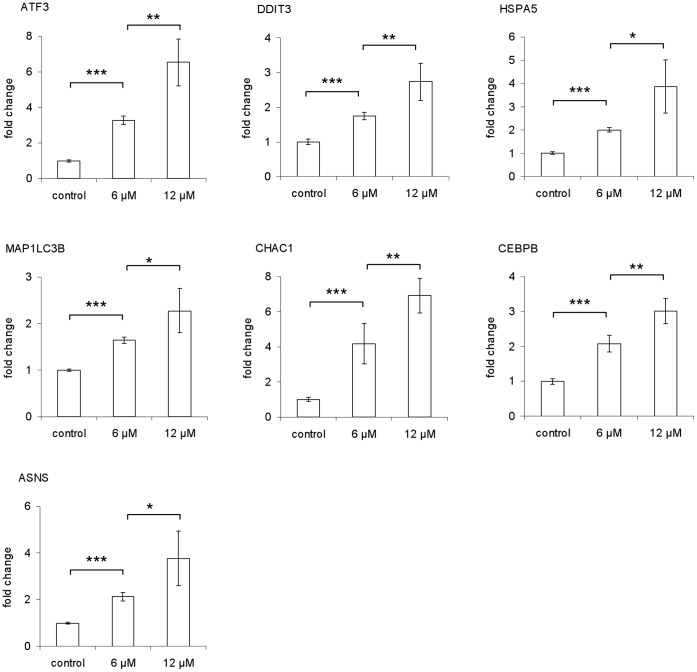
Induction of ER stress response genes by CTet in MDA-MB-231 cells. RT-qPCR assays were performed in triplicate. The data are presented as means of two independent experiments ± SD (*p<0.05; **p<0.01; ***p<0.001).

**Figure 2:**
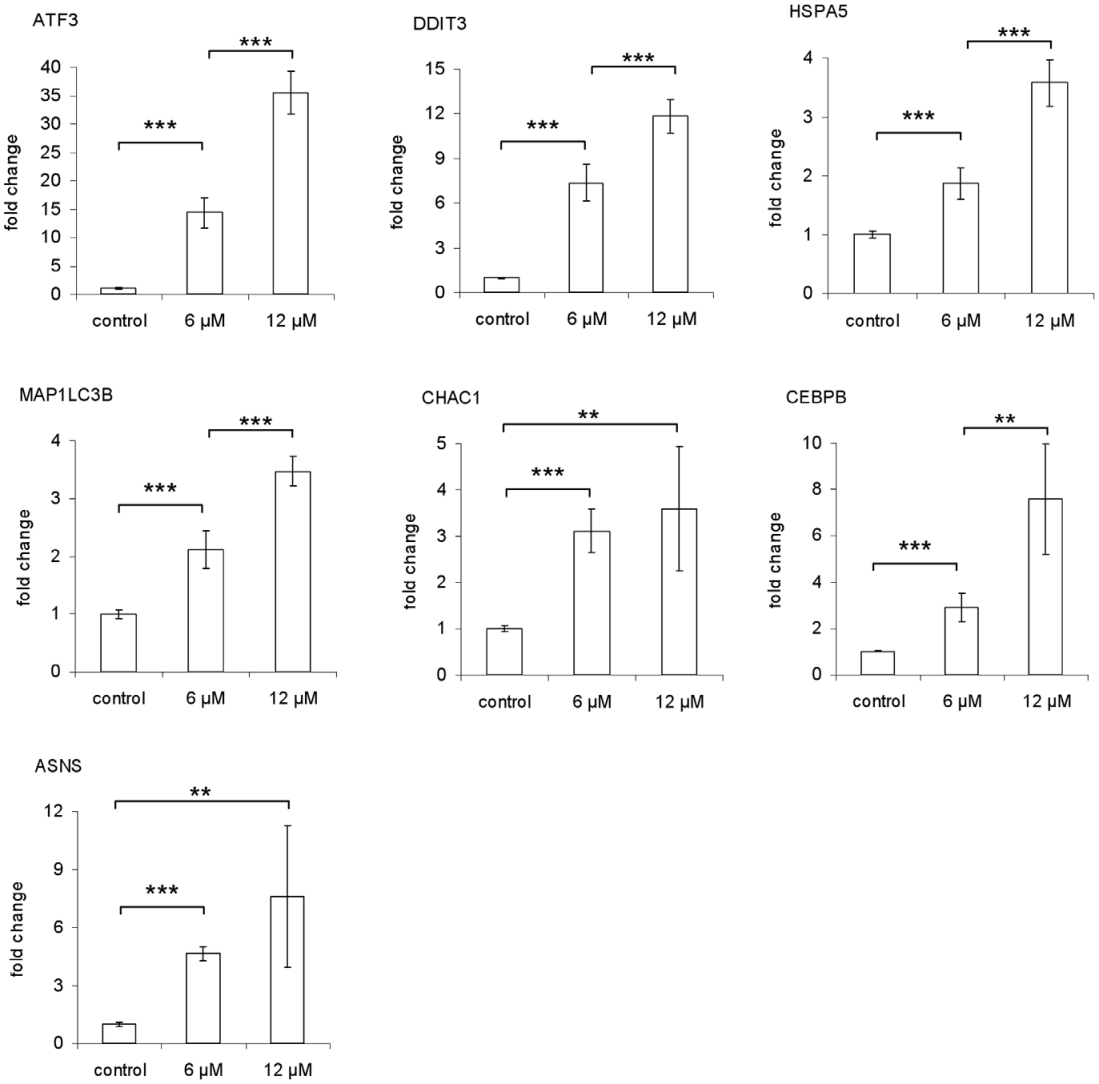
Induction of ER stress response genes by CTet in MCF-7 cells. RT-qPCR assays were performed in triplicate. The data are presented as means of two independent experiments ± SD (**p<0.01; ***p<0.001).

MCF-7 and MDA-MB-231 cells were treated with CTet for 24 h, as described in materials and methods, before extraction of total RNA. The mRNA from the entire panel of ER stress marker genes was significantly (p<0.01) increased at 24 h after addition of CTet (both 6 µM and 12 µM) in MDA-MB-231 (Fig. 1) and MCF-7 cells (Fig. 2). Moreover, the gene induction appeared generally dose-dependent, and the extent of up-regulation was sometimes higher than microarray data (Table 1). This may be due to the greater sensitivity of the RT-qPCR technique compared to microarray analysis.

### CTet Induces Xbp-1 Splicing

**Figure 3:**
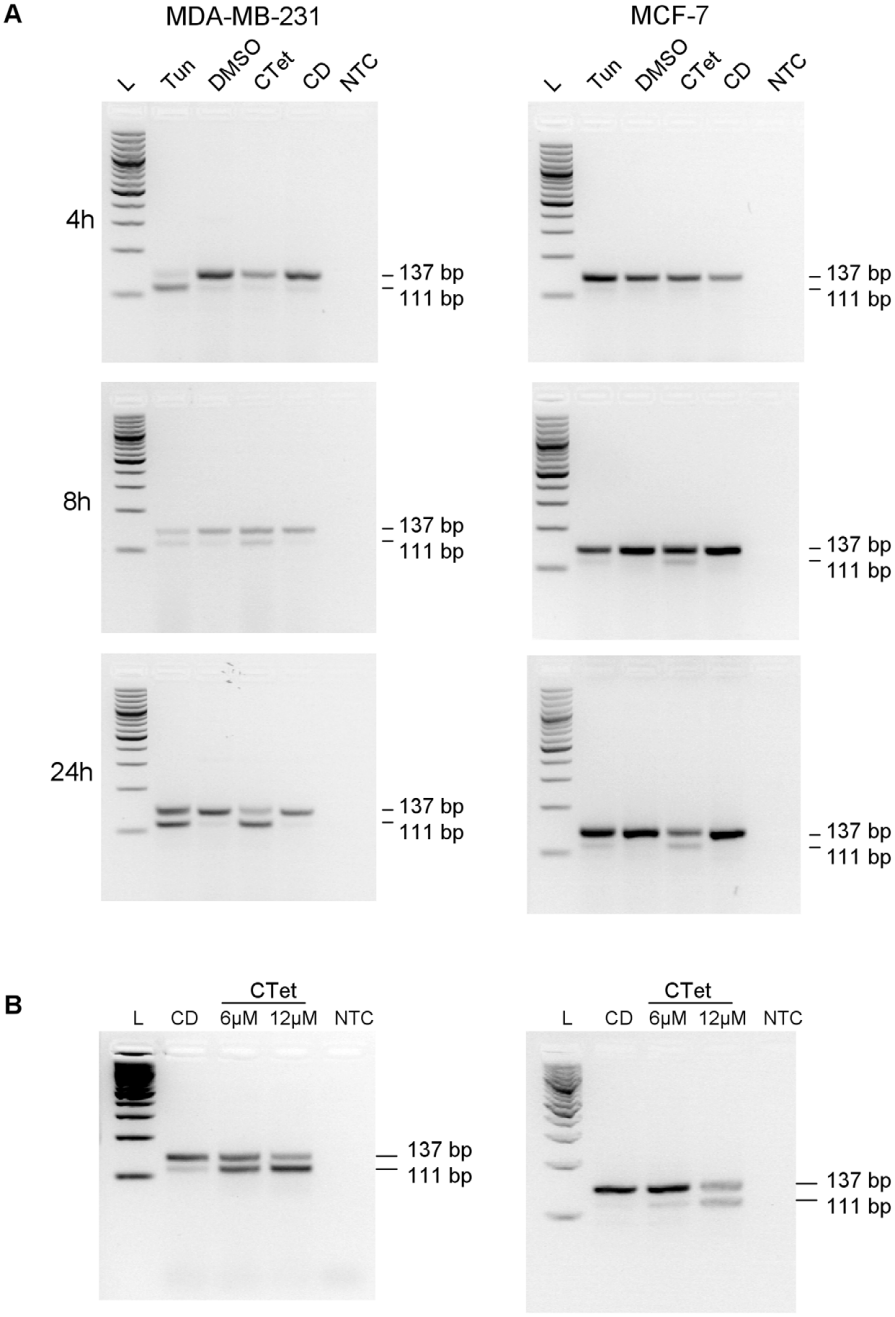
Alternative splicing of Xbp-1 mRNA in CTet-treated cells. MDA-MB-231 (left) and MCF-7 cells (right) were treated with CTet or tunicamycin, and total RNA was reverse transcribed and analyzed by PCR for detection of alternative spliced forms, as described in methods. (A) Cells were treated with 12 µM CTet or 2 µg/ml tunicamycin for 4, 8 and 24 h. Cells treated with γ-cyclodextrin and DMSO were used as negative controls for CTet and tunicamycin treatment, respectively. (B) Cells were treated with 6 µM and 12 µM CTet for 24 h. Cells treated with γ-cyclodextrin were used as control. One representative experiment is shown for each cell line. L, 100 bp DNA ladder; Tun, tunicamycin; CD, γ-cyclodextrin; NTC, no template control.

One effect of ER stress is the activation of transcription factor Xbp-1 by unconventional splicing of its mRNA mediated by IRE1, leading to the elimination of a 26-nucleotide intron [6]. We determined whether the Xbp-1 transcript was subjected to this splicing in MCF-7 and MDA-MB-231 cells treated with CTet 12 µM. The treated cells were harvested at 4 h, 8 h and 24 h, and Xbp-1 splicing was monitored as described in methods. For each time point, cells treated with tunicamycin were considered as positive control. In fact, tunicamycin is known to inhibit glycosylation of newly synthesized proteins, inducing ER stress and Xbp-1 mRNA splicing [14]. γ-Cyclodextrin-treated cells and DMSO-treated cells were used as negative controls for CTet and tunicamycin treatment, respectively. The PCR products of unspliced and spliced forms had an electrophoretic mobility compatible with their predicted length of 137 and 111 bp, respectively (Fig. 3). The amplicons were excised from the gel, purified and sequenced. The obtained sequences matched with human Xbp-1 sequence present in GenBank, confirming the specificity of the assay (not shown). In MDA-MB-231 cells, tunycamicin induced early (4 h) Xbp-1 splicing while this response was delayed (8 h) in CTet-treated cells. The MCF-7 cells appeared less susceptible to Xbp-1 splicing; in fact, the spliced/unspliced ratio was always lower compared to MDA-MB-231 cells. However, the spliced Xbp-1 form was evident for tunicamycin- and CTet-treatment after 8 h (Fig. 3A). The experiments were also repeated with cells treated for 24 h with 6 µM and 12 µM of CTet, to test the same conditions used for microarray experiments. The results confirmed the data obtained at 24 h; moreover, the smaller PCR fragment of 111 bp associated with IRE1-mediated splicing was shown to increase in a dose-dependent manner (Fig. 3B).

### CTet Treatment Induces Accumulation of Ubiquitinated Substrates

**Figure 4:**
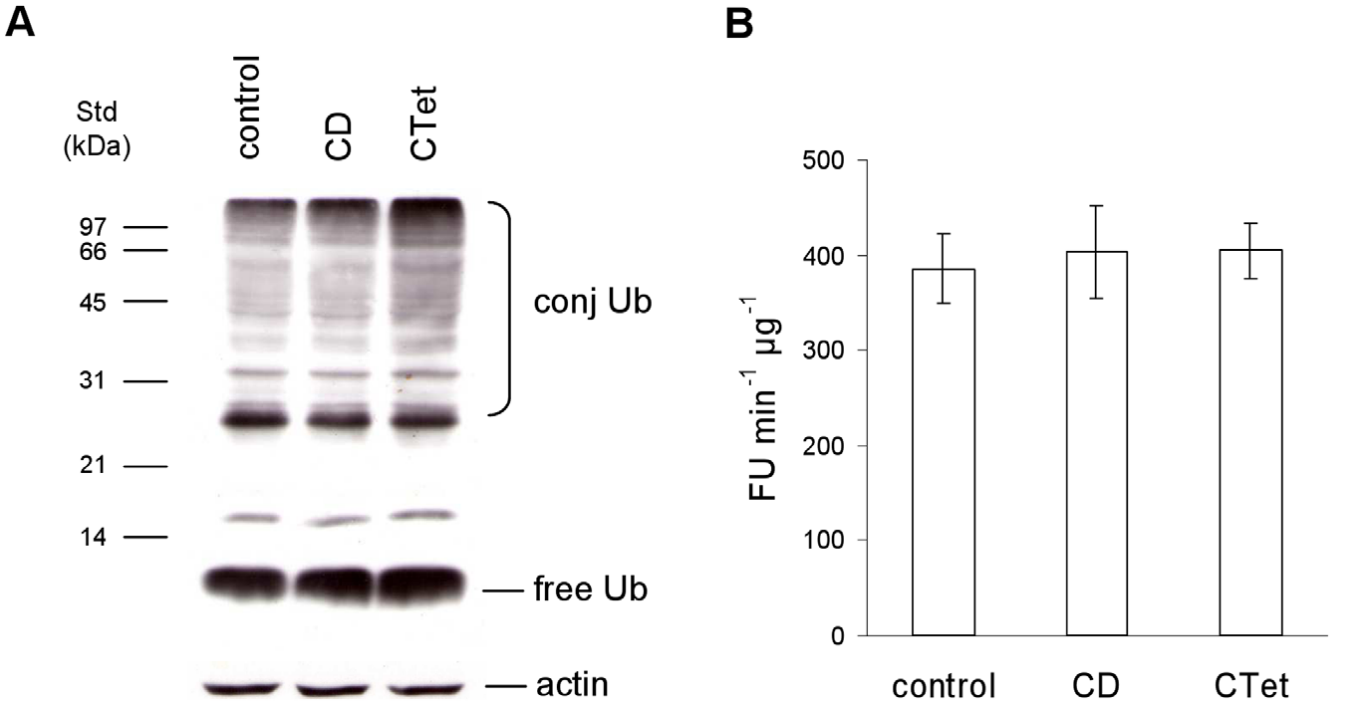
Ubiquitin pools and 20S proteasome activity in CTet-treated cells. (A) Whole protein extracts (15 µg) were obtained from MDA-MB-231 cells left untreated (control) or incubated with the vehicle (CD) or 8 µM CTet for 24 h. Protein extracts were separated by SDS-PAGE onto 12% gels, blotted onto nitrocellulose membrane and immunostained with an antibody against ubiquitin. Molecular weight markers are indicated on the left. Actin was stained as a loading control. (B) To assay 20S proteasome activity, 5 µg of cell extracts, obtained from control, vehicle-treated and CTet-treated MDA-MB-231 cells, were incubated in the presence of 200 µM sLLVY-NH-Mec at 37°C. The breakdown of the fluorigenic peptide was monitored using a fluorescence microplate reader. Data are presented as the means ± SD of three independent experiments. FU, fluorimetric units.

Western blot analysis of free and conjugated ubiquitin (Ub) showed that CTet caused a 2.5- and 2-fold increase in the levels of ubiquitinated proteins, compared to control and vehicle-treated cells, respectively (Fig. 4A). A slight 1.5-fold increase was detected in the amount of the free ubiquitin monomer (Fig. 4A). On the whole these observations suggest that the ubiquitin-proteasome system may be compromised or the amount of misfolded proteins in the cytosol exceed the capacity of the cell’s degradation system. One of the main mechanisms accounting for the accumulation of ubiquitin conjugated proteins is a reduction or a suppression of proteasome activity. Hence, we measured the activity of the proteasome using a fluorogenic substrate. This experiment did not show any significant differences between untreated, vehicle-treated or CTet-treated cells (Fig. 4B), indicating that the proteasome core complex is unaffected and therefore unlikely to be responsible for ubiquitinated substrate accumulation.

### Evaluation of Protective Autophagy/Autophagic Cell Death

**Figure 5:**
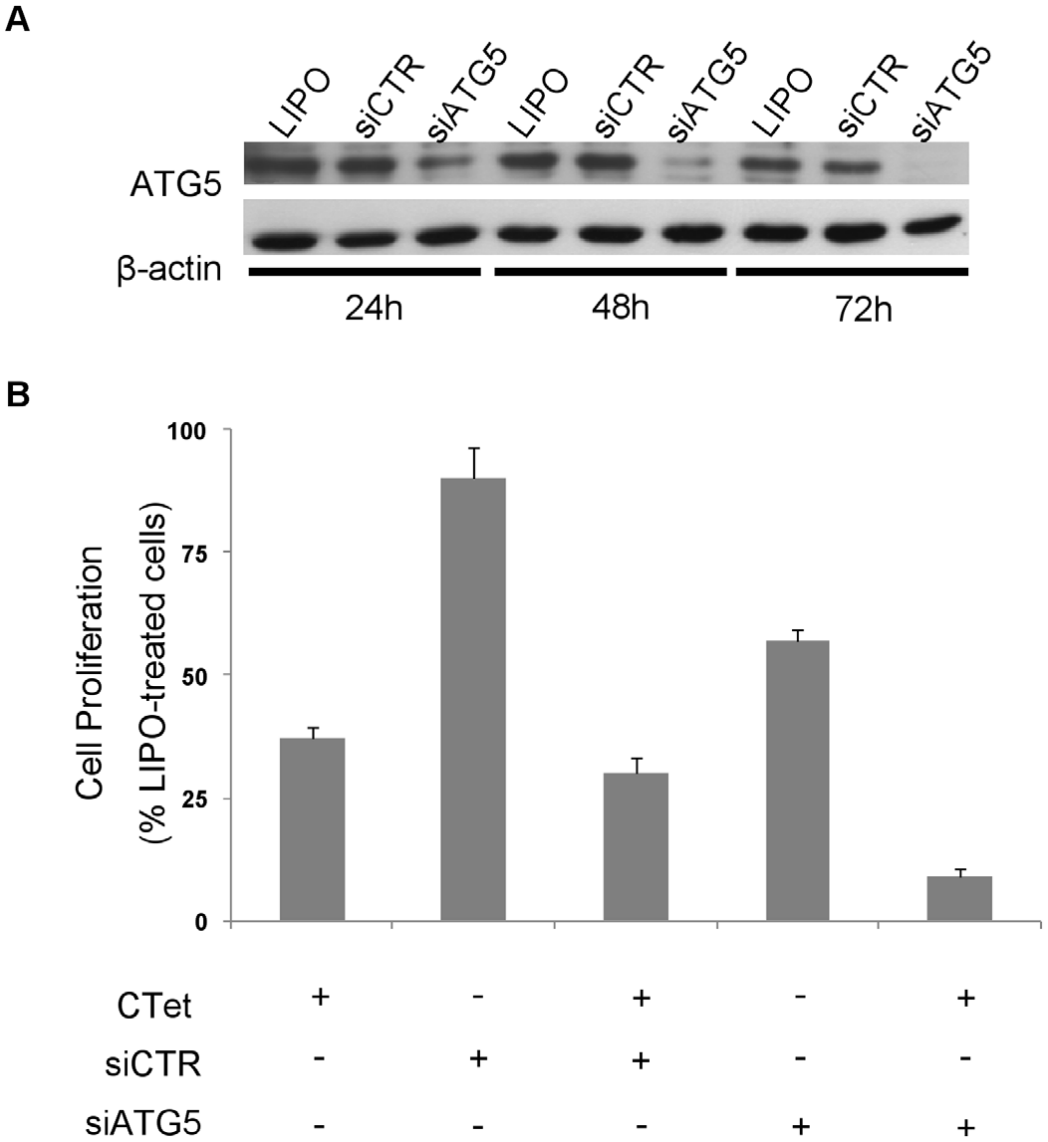
Silencing of ATG5 gene. MDA-MB-231 cells were transfected with siRNA targeting ATG5 gene (siATG5) and control siRNA as described in methods. (A) Time-dependent decrease in ATG5 protein in siATG5-transfected cells. (B) CTet activity in ATG5-depleted cells. The ATG5 gene silencing greatly increases cell sensitivity to CTet compared to control siRNA- or non-transfected cells. Data are means ± SEM of three experiments.

The autophagy induction in CTet-treated breast cancer cells has been demonstrated as co-localization of the autophagosome marker LC3b and lysosome marker LAMP2a by immunofluorescence [2]. In this study, the role of CTet-induced autophagy was initially investigated by depleting the ATG5 gene, a member of the autophagy-specific ATG family genes, which is involved in the early steps of autophagosome formation. Transfection of MDA-MB-231 cells with a siRNA targeting ATG5 (siATG5) resulted in a marked and time-dependent decrease in ATG5 protein, compared to control siRNA or the carrier alone (Fig. 5A). ATG5-depleted cells showed a remarkable increase in their sensitivity to CTet compared to control siRNA- or non-transfected cells (Fig. 5B), thus suggesting that autophagy could represent a safeguard mechanism initially activated by breast cancer cells to counteract CTet-mediated cellular stress.

The role of autophagy during CTet treatment was further investigated by inhibiting AVOs formation at different time points with bafilomycin A1 and 3-methyladenine (3-MA). MDA-MB-231 and MCF-7 cells were treated with increasing concentration of CTet for 72 h. Bafilomycin (1 nM) and 3-MA (1 mM) were added in culture medium at different time point (0 h, 24 h and 48 h after beginning CTet treatment). AVOs formation and cell viability were evaluated at the end of treatments by acridine orange staining and MTS assay, respectively.

Bafilomycin A1 was shown to efficiently inhibit AVOs formation in both MDA-MB-231 and MCF-7 cells when added simultaneously to CTet (T0, not shown) and 24 h after starting of CTet treatment (Figure S1), whereas 3-MA was shown to efficiently inhibit AVOs formation only when added at T0 (Figure S2).

**Figure 6:**
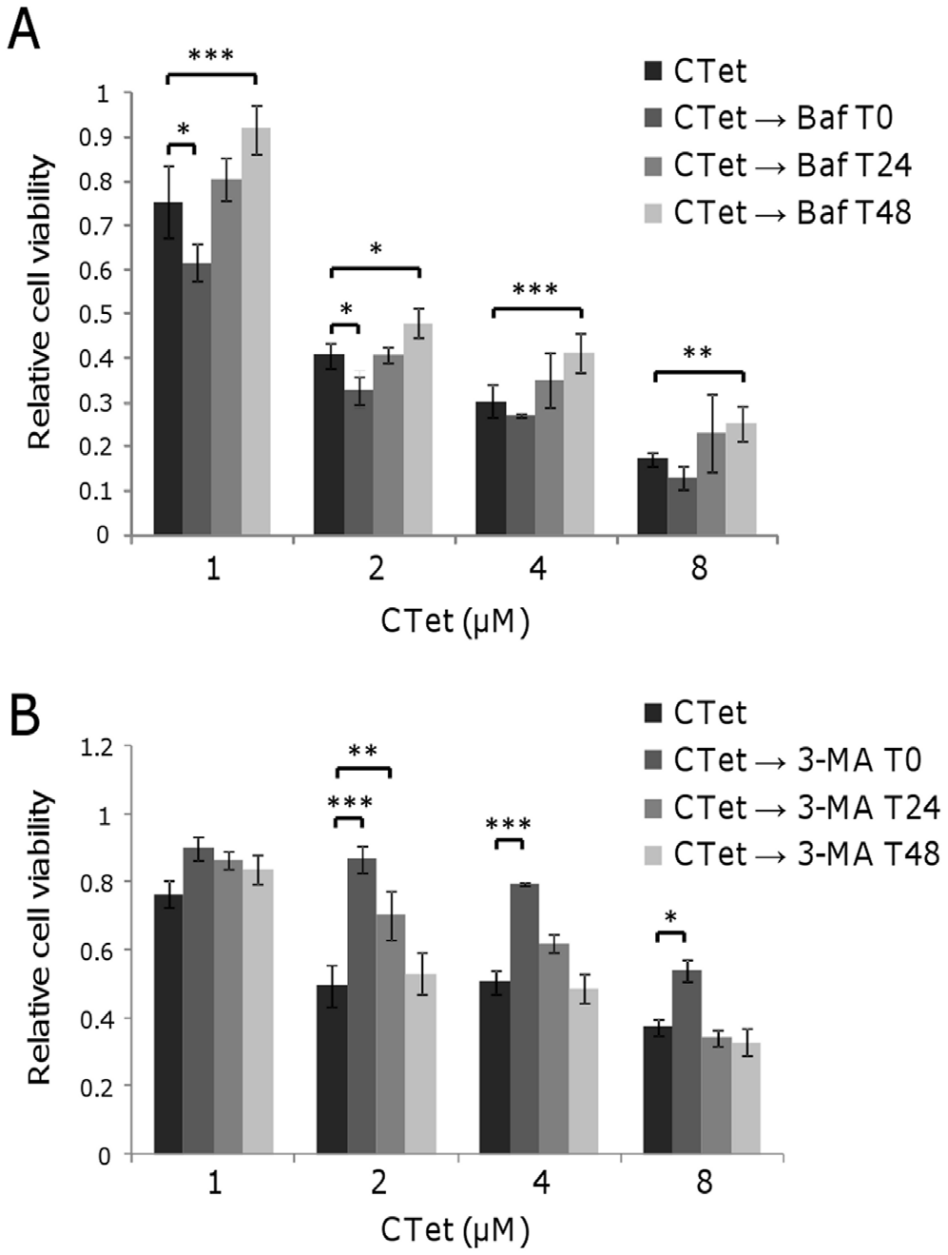
Effect of autophagy inhibition in CTet-treated breast cancer cells. MDA-MB-231 cell were treated with increasing concentration of CTet and autophagy was pharmacologically inhibited at indicated time with 1 nM bafilomycin A1 (A) and 1 mM 3-MA (B). Inhibiting autophagy after 48 h of treatment significantly reduced CTet activity, indicating the role of autophagy in cell death. Using 3-MA as autophagy inhibitor, CTet activity significantly decreased in MDA-MB-231 cells when autophagy inhibition occurred at T0. Data are expressed as relative cell viability normalized to bafilomycin- or 3-MA-treated cells. Data are means ± SD of at least two experiments performed in triplicate. *p<0.05; **p<0.01; ***p<0.001.

The CTet activity in MDA-MB-231 cells increased when autophagy inhibition with bafilomycin occurred at early treatment times, while it significantly decreased when autophagy inhibition occurred after 48 h of treatment (Fig. 6A). These results suggest that CTet may cause an autophagic protective response at early time points of treatment, whereas CTet-treated cells undergo autophagic cell death after 48 hours. On the other hand, using 3-MA as autophagy inhibitor, CTet activity significantly decreased in MDA-MB-231 cells when autophagy inhibition occurred at T0 (Fig. 6B).

Autophagy inhibition in MCF-7 cells did not significantly reduce CTet activity, with the exception of 32 µM CTet, using 3-MA as inhibitor (Figure S3). This is probably because autophagy appears to occur transiently following exposure solely to the highest CTet concentration in immunofluorescence assay [2].

### Apoptosis/Necrosis Evaluation

Induction of apoptosis was evaluated at 24 h, 48 h and 72 h by fluorescence microscopy in cells treated with 8 µM CTet and stained with DAPI. Cells treated with paclitaxel (a mitotic inhibitor and inducer of apoptosis) were used as positive control. While nuclear fragmentation was visible in cells treated with paclitaxel, such morphologic features were not evident in cells treated with CTet (Figure S4).

**Figure 7:**
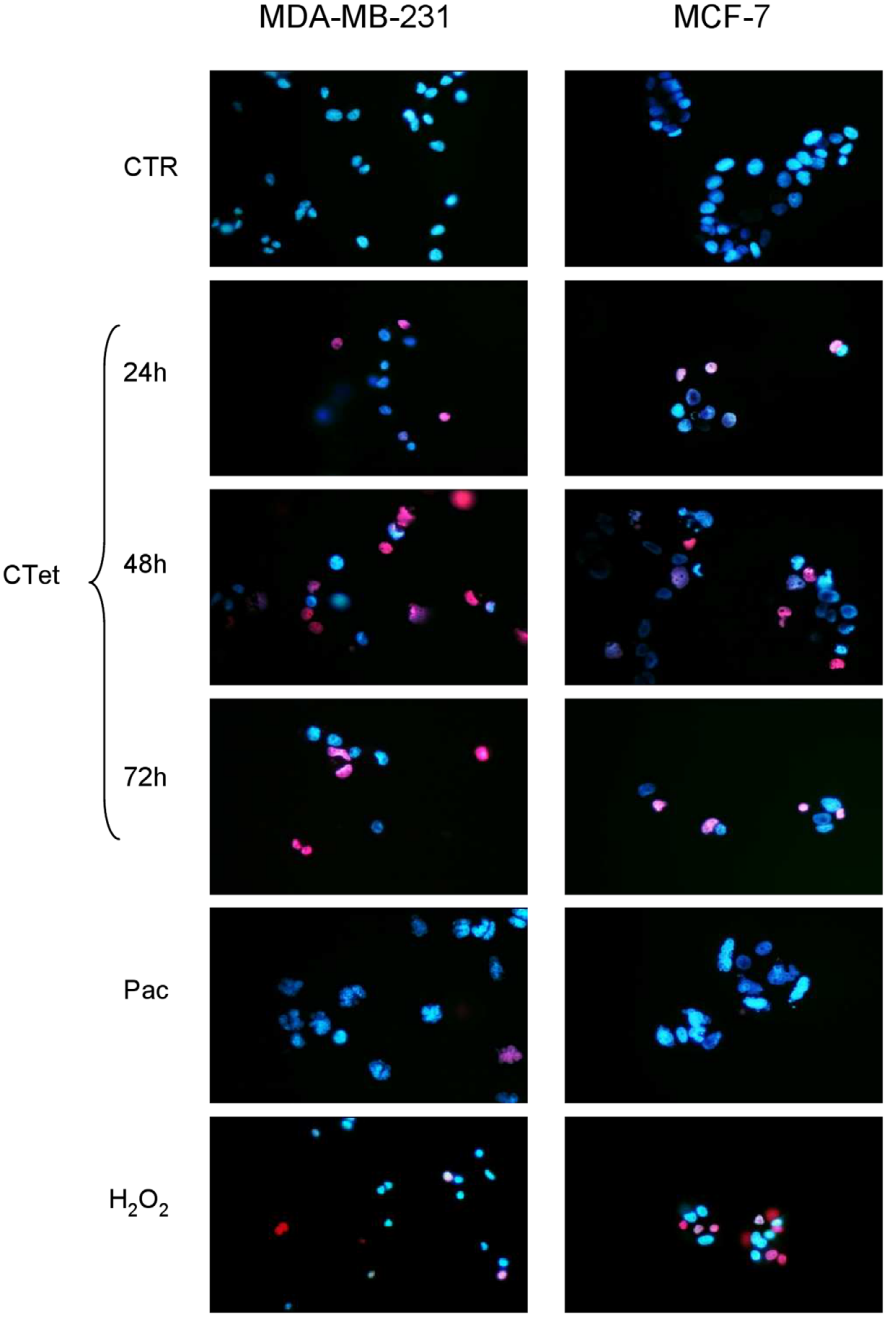
Evaluation of apoptotic/necrotic processes. MDA-MB-231 (left panel) and MCF-7 (right panel) were treated with 8 µM CTet for 72 h and stained with PI/Hoechst for necrosis/apoptosis evaluation. Paclitaxel was used as a positive control for apoptosis, H_2_O_2_ was used as a positive control for necrosis. Results show that CTet induced necrosis (PI-positive nuclei, red) starting from 24 h in both CTet-treated cell lines, without any evidence of apoptosis induction (nuclear fragmentation).

In order to investigate non-apoptotic cell death, necrosis was evaluated at 24 h, 48 h and 72 h by fluorescence microscopy in cells treated with CTet and stained with Hoechst/PI. Cells treated with paclitaxel were used as positive control for apoptosis, while cells exposed to H_2_O_2_ were used as positive control for necrosis. PI-positive nuclei (i.e. necrotic cells) were detected from 24 h in both CTet-treated cell lines, while no morphological features typical of apoptosis, such as nuclear fragmentation, were visible (Fig. 7). These results were also confirmed on MDA-MB-231 cells using Annexin V–PI staining followed by flow cytometry. The amount of annexin V+/PI− CTet-treated cells was always below 10%, while PI+ cells were between 30% and 70%, depending from the CTet dose and time of treatment (Figure S5).

Taken together, these results suggest that cells treated with CTet die by necrosis which follows autophagic processes.

### Induction of Reactive Oxygen Species (ROS)

Since intracellular ROS can be generated under ER stress conditions, and since ROS can activate autophagy and autophagic cell death [15], the production of ROS during CTet treatment was examined at 24 h, 48 h and 72 h. The results did not show an evident increase in the intracellular production of ROS, compared to untreated controls, in MDA-MB-231 (Figure S6A) or in MCF-7 (Figure S6B) cell lines.

## Discussion

I3C and its metabolites have been shown to have a broad range of antitumor activities in human cancer cells [1]. In a previous investigation we demonstrated that the I3C derivative CTet is able to induce autophagy, and to inhibit cell proliferation in estrogen receptor-positive and triple negative breast cancer cell lines through p21/CDKN1A and GADD45A overexpression [2].

In this study, we investigated the upstream molecular mechanisms through which CTet induced these events. The analysis of previous DNA microarray experiments in both MCF-7 and MDA-MB-231 cell lines [2] allowed us to identify, among the significantly up-regulated genes, a subset of transcripts with roles in ER stress response and autophagy (Table 1). In particular, several chaperones and many other genes induced by the transcription factors Xbp-1, ATF4, ATF6, DDIT3/CHOP (and DDIT3/CHOP itself, which is induced by ATF4) were identified. The mRNA level of ATF4 (induced by activated eIF2α) and genes considered to be ER stress sensors (such as ATF6, IRE1) were not significantly changed in microarray experiments. This might be explained by the fact that the analysis was performed after 24 h treatment and some early gene induction may have been missed. Moreover, the up-regulation of some ER stress markers may not have been detected because of low fold change or low statistical significance. This was the case of PERK/EIF2AK3, HERPUD1/HERP, PPP1R15A/GADD34, and HSPA5/BiP/GRP78 (see Results).

Among the methods for monitoring ER stress, the detection of targets downstream the three proximal ER stress sensors IRE1, PERK and ATF6, as well as detection of spliced Xbp-1 mRNA, are considered robust approaches [13]. Thus, to confirm the induction of ER stress response, the expression of six ER stress marker genes and one autophagy-related gene was analyzed by RT-qPCR in MCF-7 and MDA-MB-231 cell lines. In addition, the Xbp-1 splicing induced by activated IRE1 was also monitored, showing the appearance of the spliced Xbp-1 form after 8 h CTet treatment in both cell lines. This delay in the induction of Xbp-1 splicing could be explained by the poor solubility (and consequently slow bioavailability) of CTet, the uptake of which may be mediated by phagocytic processes, more active in MDA-MB-231 cells [16]. Taken together, these results strongly support the hypothesis that CTet induces an ER stress response in either MCF-7 or MDA-MB-231 cells.

CTet treatment has also shown to induce accumulation of ubiquitinated substrates, which has been related to ER stress [17]. Since ER stress can be triggered by proteasome inhibitors [18], we evaluated the 20S proteasome activity in CTet-treated cells. The results clearly showed that CTet does not interfere with proteasome activity, therefore excluding this mechanism as a contributor to ER stress induction.

The presence of a faint sXbp-1 band in MDA-MB-231 (but not in MCF-7) control cells may be due to a basal activation of ER stress response in these cancer cells. In fact, ER stress response may be important for the growth of tumors under the stressful conditions commonly encountered by most solid tumors (i.e. hypoxia or glucose deprivation) [12]. This feature, together with the fact that the extent of Xbp-1 splicing was greater in MDA-MB-231 cells than in MCF-7 cells, may help to explain the previously reported greater (p<0.05) susceptibility to CTet of MDA-MB-231 cells compared to MCF-7 cells (IC_50_ at 72 h = 1.00±0.01 µM and 1.32±0.03 µM, respectively) [2]. Indeed, it could be inferred that MDA-MB-231 cells are more prone to activate the IRE1/Xbp-1 ER stress response branch.

It has been shown that DIM (another hydrophobic I3C derivative) can activate ER stress response in C33A cells (human cervical cancer) [19], suggesting an at least partially similar molecular effect of these I3C derivatives. On the other hand, while DIM has been shown to induce apoptosis [19], [20], our results indicate that breast cancer cells treated with CTet undergo autophagy which evolves in necrotic processes. In fact, we showed that pharmacological inhibition of autophagy by bafilomycin A1 and 3-MA significantly decreased CTet activity, indicating that CTet is able to induce autophagy-related cell death, although the effect of bafilomycin and 3-MA was evident when autophagy inhibition occurred at T 48 and at T 0, respectively (Fig. 6). These differences may reflect the different mechanisms of action and/or toxicities of these molecules. Further studies are needed to fully characterize the autophagic processes triggered by CTet.

The ER stress response usually implies the activation of autophagic protective processes and, if the cell does not recover from the stress, the induction of cell death primarily through apoptosis [21]. However, it has also been shown that prolonged ER stress can result in necrosis-like cell death associated with autophagy in cells with impaired apoptosis [22] and that inhibition of autophagy during ER stress results in reduced cell death in apoptosis-deficient cells [22]. Considering that the model cell lines that we used in this study were the p53-mutant MDA-MB-231 [23] and the caspase-3 deficient MCF-7 [24], our data appear to be in agreement with these observations. Therefore, it is highly plausible that CTet, through ER stress induction, elicit necrosis-like cell death in breast cancer cell lines.

Since ER stress and autophagy can be related to ROS generation, the production of intracellular ROS during CTet treatment was also investigated. It was found that Ctet does not induce a ROS production or, at least, that the observed ER stress response involved the activation of mechanisms sufficient to scavenge ROS, such as induction of SOD2, HMOX1 and TXNRD1 genes (Table 1), or the autophagic processes themselves, which can suppress cellular ROS level by eliminating ROS-producing compartments.

**Figure 8:**
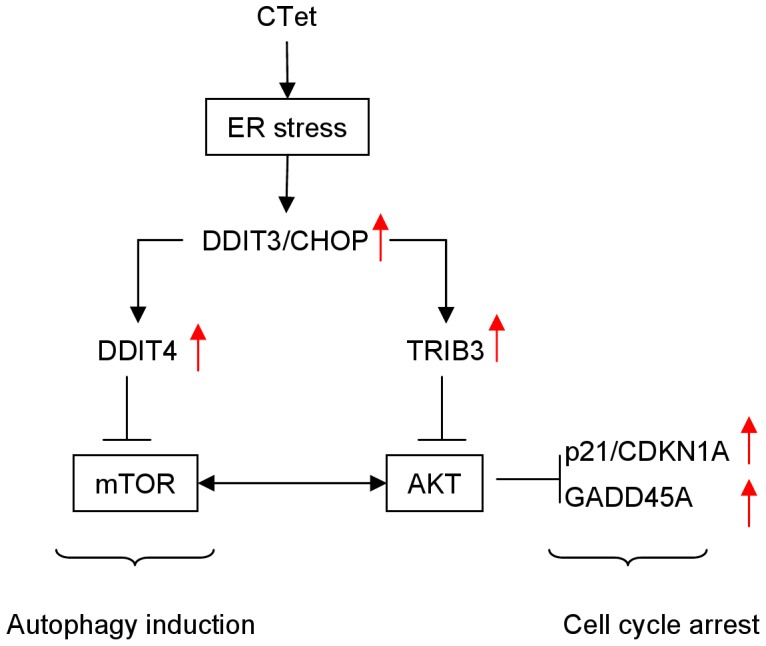
Signaling pathways affected by CTet. Simplified scheme illustrating the mechanism through which CTet could lead to autophagy induction and previously observed [2] p21 and GADD45A overexpression. Arrows indicate gene up-regulation.

In a previous investigation we showed that CTet activity is in part mediated by the inhibition of Akt phosphorylation [2]. The PI3K/Akt signal transduction pathway, involved in cell survival and proliferation, is interconnected with the mTOR pathway. In fact, Akt can phosphorylate and activate the mTOR-containing complex mTORC1 directly or indirectly through the actions of the TSC1/TSC2 complex [25]; moreover, the second mTOR-containing complex, mTORC2, contributes to phosphorylation and activation the Akt kinase [26]. Thus, mTOR can play a role both upstream (mTORC2) and downstream (mTORC1) of Akt. The kinase mTOR is also a critical regulator of autophagy, with the activated mTOR acting as an autophagy inhibitor [26]. Among the up-regulated genes listed in Table 1, it is noteworthy the presence of DDIT4/REDD1, an inhibitor of the mTOR pathway acting downstream of AKT and upstream of TSC2 [27], and TRIB3, a negative regulator of Akt [28]. Hence, ER stress, through the induction of DDIT4 and TRIB3, might explain the inhibition of Akt activity, and previously observed p21 and GADD45A up-regulation [2] (Fig. 8). It is therefore highly likely that multiple mechanisms (ER stress response, Akt signaling and mTOR pathway) are operating to inhibit the growth of human breast cancer cells.

## Materials and Methods

### Cell Cultures

The human breast carcinoma estrogen receptor positive (MCF-7) and triple negative (MDA-MB-231) cell lines were purchased from the American Type Cell Culture Collection and were cultured in DMEM supplemented with 10% Fetal Calf Serum (FCS), 2 mM L-glutamine, 10 g/L Non-Essential Amino Acid, 50 mg/L streptomycin, 1000 U/L penicillin. MCF-7 cells were also supplemented with 10 mg/L insulin. All cell culture reagents were purchased from Sigma-Aldrich (St. Louis, MO).

### Reagents

CTet was synthesized as described [2], [29], [30]. The identity and purity of CTet were assessed by HPLC/MS and ^1^H NMR analyses, and are according to the literature [29]. CTet was formulated as reported previously [29]. In all experiments, 10 µl of the concentrated agent were added per ml of cell culture medium. The vehicle control was 10 µl of aqueous solution of γ-cyclodextrin.

Tunicamycin was purchased from Sigma-Aldrich (St. Louis, MO, USA) and dissolved in DMSO at concentrations of 2 mg/ml for storage.

### MTS Assay

Cells were plated at a density of 5×10^3^ cells/well in 96-well plate, incubated at 37°C overnight and treated in triplicate with increasing concentration of CTet and/or Bafilomycin A1 and 3-MA (Sigma-Aldrich). After 72 h of treatment, cell viability was evaluated using CellTiter 96® Aqueous Non-Radioactive Cell Proliferation Assay (Promega) based on the ability of viable cells to convert a soluble tetrazolium salt [3-(4,5-dimethylthiazol-2-yl)-5-(3-carboxymethoxyphenyl)-2-(4-sulfophenyl)-2*H*-tetrazolium, MTS] to a formazan product. After treatments the MTS/PMS (phenazine methosulfate, Sigma-Aldrich) reagent was added following manufacturer instructions and cell cultures were incubated at 37°C for 4 h. Absorbance was recorded on a Microplate Reader (Benchmark, Bio-Rad) at 492 nm and at a reference wavelength of 630 nm. The results were expressed as relative viable cells compared to control (vehicle only).

### Real Time-quantitative PCR (RT-qPCR)

Breast cancer cells were plated in 6-well culture plates at a density of 1.5×10^5^ cells/well and cultured overnight at 37°C in a humidified incubator with 5% CO_2_. Cells were then treated with 6 µM or 12 µM of CTet (and vehicle control). Viable cells were counted by trypan blue dye exclusion, washed in phosphate buffered saline, and the cell pellets were stored at −20°C in 300 µl of RNA-later solution (Sigma-Aldrich) for successive RNA extraction. Total RNA was reverse transcribed using SuperScript First Strand Synthesis System (Invitrogen) with oligo-dT priming.

RT-qPCR primers were designed using Primer Express software (Applied Biosystems) and are listed in Table 2. All primer pairs spanned an intron or were designed on 2 different exons, with the exception of MAP1LC3B and CEBPB primers. To confirm the primer sequences specificity, a BLAST search against the “human genomic plus transcript" database was performed.

**Table 2:** Primer sequences for SYBR green RT-qPCR.

Target mRNA	Accession number	Forward primer (5′–3′)	Reverse primer (5′–3′)	Amplicon lenght (bp)	Ref.
MAP1LC3B	NM_022818	AAACGGGCTGTGTGAGAAAAC	TGAGGACTTTGGGTGTGGTTC	83	
DDIT3	NM_001195053	GGAGCATCAGTCCCCCACTT	TGTGGGATTGAGGGTCACATC	101	[35]
CHAC1	NM_024111	TGGTGACGCTCCTTGAAGATC	GCACTGCCTCTCGCACATT	111	
ATF3	NM_001674	GCCATCCAGAACAAGCACCT	GGCTACCTCGGCTTTTGTGAT	105	
HSPA5	NM_005347	CCCCGAGAACACGGTCTTT	CAACCACCTTGAACGGCAA	101	
CEBPB	NM_005194	CGAAGTTGATGCAATCGGTTT	TTAAGCGATTACTCAGGGCCC	101	
ASNS	NM_133436	ATTGGCTGCCTTTTATCAGGG	TGTCTTCCATGCCAATTGCA	121	
GAPDH	NM_002046	CCATGTTCGTCATGGGTGTG	GGTGCTAAGCAGTTGGTGGTG	91	

MAP1LC3B (microtubule-associated protein 1 light chain 3 beta); DDIT3 (DNA-damage-inducible transcript 3); CHAC1 [ChaC, cation transport regulator homolog 1 (E. coli)]; ATF3 (activating transcription factor 3); HSPA5 [heat shock 70 kDa protein 5 (glucose-regulated protein, 78 kDa)]; CEBPB [CCAAT/enhancer binding protein (C/EBP), beta]; ASNS (asparagine synthetase); GAPDH (glyceraldehyde-3-phosphate dehydrogenase).

RT-qPCR was performed in triplicate in a final volume of 20 µl using SYBR green PCR master mix (Applied Biosystems, Foster City, CA), 200 nM primers, in a RotorGene 6000 instrument (Corbett life science, Sydney, Australia), with the following amplification conditions: 95°C for 10 min, 40 cycles at 95°C for 15 s and 60°C for 50 s. A triplicate non-template control was included for each primer pair reaction. At the end of each run, a melting curve analysis from 55°C to 90°C was performed to ensure the absence of primer dimers or non-specific products. GAPDH (glyceraldehyde-3-phosphate dehydrogenase) was used as a reference gene, and vehicle control-treated cells were used as calibrator. Relative mRNA expression was calculated using the comparative quantification application of the RotorGene 6000 software. All RT-qPCR expression values were determined from 2 independent biological experiments.

### Detection of Xbp-1 mRNA Splicing

Detection of Xbp-1 mRNA splicing was done by PCR amplification of cDNA prepared as described above, using primers designed upstream (Xbp1-F 5′-GGGAATGAAGTGAGGCCAGT-3′) and downstream (Xbp1-R 5′-TGAAGAGTCAATACCGCCAGA-3′) of the 26-nucleotides spliced sequence. Cells treated with 2 µg/ml tunicamycin were used as positive control for Xbp-1 splicing; in this case, control cells were treated with DMSO. The PCR was performed in a final volume of 50 µl, using 200 nM of each primer, 0.2 mM dNTPs, 2 mM MgCl_2_ and 1 unit of Hot-Rescue DNA Polymerase (Diatheva srl), in a GeneAmp PCR System 2700 (Applied Biosystems), with the following conditions: 94°C for 7 min, 32 cycles at 94°C for 10 s, 57°C for 5 s and 72°C for 15 s, and a final step at 72°C for 30 s. The amplification product of the unspliced Xbp-1 mRNA was 137 bp, while the amplification product of the spliced form was 111 bp. PCR products were separated on 3.5% high-resolution MetaPhor agarose (Cambrex) gel and visualized with GelRed DNA stain (Diatech). A 100-bp double-stranded DNA ladder (MBI Fermentas) was included on the gels as a size standard.

### Detection of Acidic Vesicular Organelles (AVOs)

As marker of autophagy, the appearance and volume of AVOs were analyzed by acridine orange staining [31]. Briefly, 5×10^5^ MDA-MB-231 and MCF-7 cells were seeded in 60 mm diameter dish, incubated overnight, treated with 8 µM CTet and incubated for 72 h. At the indicated time points, 1 nM bafilomycin or 1 mM 3-MA were added. Cells were then incubated in serum-free medium containing 1 µg/ml acridine orange for 15 minutes at 37°C. The acridine orange was removed and fluorescent micrographs were taken using a fluorescent microscope (Blue excitation filter). The cytoplasm and nucleus of the stained cells fluoresced bright green, whereas the acidic autophagic vacuoles fluoresced bright red.

### siRNA Transfections

A siRNA directed against the target sequence 5′-CAUCUGAGCUACCCGGAUA-3′ within the open reading frame of ATG5 mRNA (GeneBank accession n. NM_004849) was selected using the online siMAXTM design tool (www.eurofinsdna.com). A control siRNA, made of a scrambled sequence with no significant homology to any known human mRNA, was included. siRNAs were manufactured by Eurofins MWG Operon (Ebersberg, Germany) as pre-formed and purified duplexes, made of 19 bp-long RNA oligonucleotides with two extra-thymidine bases forming a 3′ overhang on both strands. Each siRNA was resuspended in sterile RNase-free water, diluted to the appropriate stock solution (5 µM) and stored at −20°C until use.

The day before transfection, MDA-MB-231 cells were seeded in 6/well plates at a density of 1×10^5^ cells per well. A given amount of each siRNA was mixed with LipofectAMINE 2000 (Invitrogen, San Giuliano Milanese, Italy) for 20 minutes at room temperature according to the manufacturer’s instructions. The mixtures were then applied to the cells in a final volume of Opti-MEM I (Invitrogen) giving a final concentration of each siRNA of 25 nmol/L. After incubation for 8 h at 37°C, DMEM-F12 supplemented with serum was added. Cells were then cultured for additional intervals (up to 72 h) at 37°C before further analysis.

In the combination experiment, siRNA-transfected cells were exposed for 72 h to 4 µM CTet. At the end of treatment, cells were trypsinized and counted in a particle counter (Coulter counter, Coulter Electronics, Luton, United Kingdom). The results were expressed as percent variation in the number of viable cells in treated compared with control cells.

### Apoptosis/Necrosis Evaluation

MDA-MB-231 and MCF-7 cells were seeded at 5×10^4^ cells per well in chamber slide and allowed to attach overnight. Cells were treated with 8 µM CTet for 24, 48 and 72 h. After treatments, cells were fixed with 4% formalin in PBS, permeated with 0.2% Triton X-100, and total DNA was stained with DAPI. To discern between necrosis and apoptosis, treated cells were directly stained with 40 µg/ml PI followed by 2.5 µg/ml Hoechst dye. Cells were viewed under a UV microscope with DAPI filter. Cells treated with Paclitaxel 1 µM for 24 h and H_2_O_2_ 3 mM for 1 h were used as positive controls for apoptosis and necrosis, respectively.

Apoptosis/necrosis was also evaluated using Annexin V–fluorescein isothiocyanate (FITC) (Becton Dickinson, Sunnyvale, CA, USA)/PI staining. Briefly, cells to be analyzed were washed twice with cold PBS, resuspended in binding buffer, and incubated (10 min at room temperature in the dark) with Annexin V–FITC. At the end of the incubation, PI was added and the cells were analyzed immediately by flow cytometry. Annexin V/PI double staining allowed for distinguishing between apoptotic (Annexin V+/PI−) and nonapoptotic cells (Annexin V+/PI+ plus Annexin V−/PI+).

### Evaluation of Proteasome Activity

The activity of the 20S proteasome was assayed in MDA-MB-231 cells using the fluorogenic substrate *N*-succinyl-Leu-Leu-Val-Tyr-7-amido-4-methylcoumarin (sLLVY-NH-Mec, Sigma-Aldrich, Italy). In brief, cells were seeded in 60 mm diameter dish at a density of 8×10^5^, incubated overnight and treated with 8 µM CTet for 24 h. Cells were then harvested by trypsinization, washed in PBS (Phospahte Buffer Saline), suspended in cold 50 mM HEPES/KOH buffer pH 7.8, containing 1 mM DTT and 0.25 M sucrose, and homogenized using a Potter-Elvehjem apparatus. The extracts were then centrifuged for 10 min at 12,000 rpm in a refrigerated Eppendorf centrifuge. Aliquots of the supernatant corresponding to 5 µg of total proteins were pre-incubated at 37°C for 5 min in 100 mM HEPES/KOH buffer, pH 7.8, containing 5 mM MgCl_2_ and 10 mM KCl (final volume 200 µl). The reaction was initiated by adding the fluorigenic substrate to a final concentration of 0.2 mM. The breakdown of the peptide was monitored for 30 min using a fluorescence microplate reader (FLUOstar OPTIMA, BMG Labtech GmbH, Offenburg, Germany) with an excitation wavelength of 355 nm and an emission wavelength of 460 nm. Proteasome activity in each sample, expressed as fluorimetric units/min, was calculated by submitting data to linear regression analysis.

### Western Immunoblotting

MDA-MB-231 cells were harvested using a scraper after 24 h-treatment with 8 µM CTet and lysed in 50 mM Tris-HCl, pH 7.8; 0.25 M sucrose, 2% (w/v) SDS, 10 mM *N*-ethylmaleimide (NEM) supplemented with the cocktail of protease [10 µg/ml leupeptin, 10 µg/ml pepstatin, 4 mM 4-(2-aminoethyl)benzenesulphonyl fluoride (AEBSF) and 1 mM phenylmethylsulphonylfluoride (PMSF)] and phosphatase (1 mM NaF, 1 mM Na_3_VO_4_) inhibitors. Lysates were boiled for 5 min, then sonicated at 100 Watts for 20 sec. Cell debris was removed by brief centrifugation (5 min at 12000×g). The total protein, in the cleared lysate, was determined according to Lowry et al. [32]. Equal amounts of whole-cell extracts were resolved by sodium dodecyl sulfate polyacrylamide gel electrophoresis (SDS-PAGE) and gels were electroblotted according to Towbin et al. [33]. The blots were probed with rabbit polyclonal anti-ubiquitin (kindly provided by Prof. A.L. Haas, Department of Biochemistry and Molecular Biology, LSU School of Medicine, New Orleans, Louisiana) and anti-actin antibodies (Sigma-Aldrich). Bands were detected using horseradish peroxidase-conjugated secondary antibody (BioRad, Hercules, CA). Peroxidase activity was revealed with the enhanced chemiluminescence detection method (ECL Plus Kit, Amersham Biosciences, Arlington Heights, IL).

### Detection of Reactive Oxygen Species (ROS)

Oxidation of non-fluorescent dihydrorhodamine 123 (DHR) to rhodamine 123 was analyzed to evaluate ROS formation after CTet treatment [34]. MDA-MB-231 and MCF-7 cells were plated in 35 mm diameter dish at 1×10^5^ cells/dish, incubated overnight and treated with 8 µM CTet. After 24, 48 and 72 h, 10 µM DHR was added in culture medium and incubated for 30 min. Cells were then washed twice with PBS and nuclei were counterstained with 2.5 µg/ml Hoechst dye. Micrographs were taken using a fluorescent microscope (Blue excitation filter for ROS and DAPI filter for nuclei). As positive control, cells were treated with 1 mM H_2_O_2_ for 1 h.

### Statistical Analysis

Statistical analysis was performed using GraphPad Prism 5 (GraphPad Software, San Diego, CA). RT-qPCR results were analyzed using unpaired t test with Welch correction, while statistical differences of cell viability data were evaluated using two-way ANOVA followed by Bonferroni post-hoc test.

## Supporting Information

Figure S1 **Bafilomycin A1 inhibits AVOs formation in both MDA-MB-231 and MCF-7 cells.** MDA-MB-231 (left panel) and MCF-7 (right panel) were treated with 8 µM CTet for 48 h and stained with acridine orange. Bafilomycin A1 (1 nM) was used during the last 24 h of treatment to inhibit AVOs formation. Micrographs were taken using a fluorescent microscope (Blue excitation filter). The cytoplasm and nucleus of the stained cells fluoresced bright green, whereas AVOs fluoresced bright red (arrows). Results show that CTet induced AVOs formation in both cell lines, efficiently inhibited by Bafilomycin. CTR, control; Baf, bafilomycin A1
(PPT)

Figure S2 **3-MA effects on AVOs formation in MDA-MB-231 and MCF-7 cells.** MDA-MB-231 (left panel) and MCF-7 (right panel) were treated with 8 µM CTet for 72 h and stained with acridine orange. 3-MA (1 mM) was added simultaneously with CTet (T0) or 24 h (T24) and 48 h (T48) after beginning CTet treatment to inhibit AVOs formation. Micrographs were taken using a fluorescent microscope (Blue excitation filter). The cytoplasm and nucleus of the stained cells fluoresced bright green, whereas AVOs fluoresced bright red. Results show that CTet induced AVOs formation in both cell lines, inhibited by 3-MA when added at T0.
(PPT)

Figure S3 **Effect of autophagy inhibition in CTet-treated MCF-7 cells.** MCF-7 cells were treated with increasing concentration of CTet and autophagy was pharmacologically inhibited at indicated time with 1 nM bafilomycin A1 (A) and 1 mM 3-MA (B). Inhibiting autophagy did not reduce CTet activity, except at the highest CTet dose, when autophagy inhibition occurred with 3-MA, indicating a minor role of autophagy in MCF-7 cell death. Data are expressed as relative cell viability normalized to Bafilomycin- and 3-MA-treated cells. Data are means ± SD of at least two experiments performed in triplicate. *p<0.05; **p<0.01; ***p<0.001.
(PPT)

Figure S4 **Evaluation of apoptotic processes.** MDA-MB-231 (upper panel) and MCF-7 (lower panel) were treated with 8 µM CTet for 24, 48 and 72 h and stained with DAPI for apoptosis evaluation. Paclitaxel was used as positive control. Results showed absence of apoptotic morphologic features (i.e. nuclear fragmentation) in both CTet-treated cell lines. CTR, control; PAC, Paclitaxel.
(PPT)

Figure S5 **Evaluation of apoptosis/necrosis by Annexin V–PI staining.** MDA-MB-231 cells were treated with 4 µM and 8 µM CTet for 48 and 72 h and double stained with Annexin V/PI. The amount of apoptotic (annexin V+/PI−) CTet-treated cells was always below 10%, while nonapoptotic CTet-treated cells (Annexin V+/PI+ plus Annexin V−/PI+) varied from 30% (4 µM CTet, 48 h treatment) to 70% (8 µM CTet, 72 h treatment).
(PPT)

Figure S6 **Detection of Reactive oxygen species (ROS).** MDA-MB-231 (A) and MCF-7 (B) cells were treated with 8 µM CTet for 24, 48 and 72 h and incubated with DHR for 30 min. Nuclei were counterstained with Hoechst dye. Oxidized-DHR fluoresced bright green, whereas nuclei fluoresced blue. Results show that CTet did not induce ROS formation neither in MDA-MB-231 (A) nor in MCF-7 (B) cell lines. As positive control, cells were treated with 1 mM H_2_O_2_ for 1 h. CTR, untreated control; γ-CD, γ-cyclodextrin.
(PPT)
