# Supplementary figures and images for: Induction of Endoplasmic Reticulum Stress Response by the Indole-3-Carbinol Cyclic Tetrameric Derivative CTet in Human Breast Cancer Cell Lines
Source: PLoS One. 2012 Aug 14;7(8):e43249. doi: 10.1371/journal.pone.0043249 (PMC3419215; doi:10.1371/journal.pone.0043249)

## Slide 1
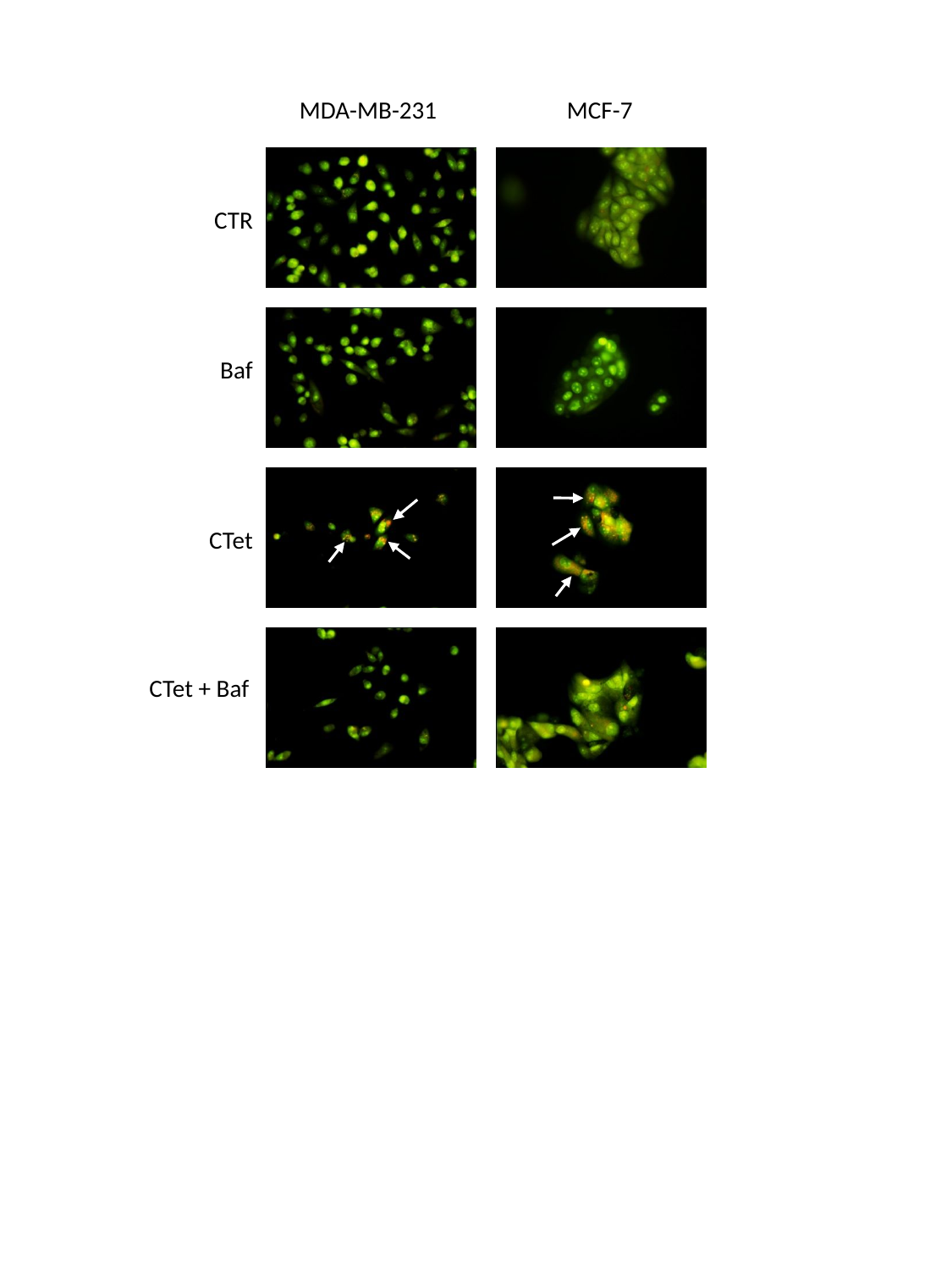

MDA-MB-231
MCF-7
CTR
Baf
CTet
CTet + Baf

Supplement: Figure S1 — Bafilomycin A1 inhibits AVOs formation in both MDA-MB-231 and MCF-7 cells. MDA-MB-231 (left panel) and MCF-7 (right panel) were treated with 8 µM CTet for 48 h and stained with acridine orange. Bafilomycin A1 (1 nM) was used during the last 24 h of treatment to inhibit AVOs formation. Micrographs were taken using a fluorescent microscope (Blue excitation filter). The cytoplasm and nucleus of the stained cells fluoresced bright green, whereas AVOs fluoresced bright red (arrows). Results show that CTet induced AVOs formation in both cell lines, efficiently inhibited by Bafilomycin. CTR, control; Baf, bafilomycin A1 (PPT) [file pone.0043249.s001.ppt]

## Slide 1
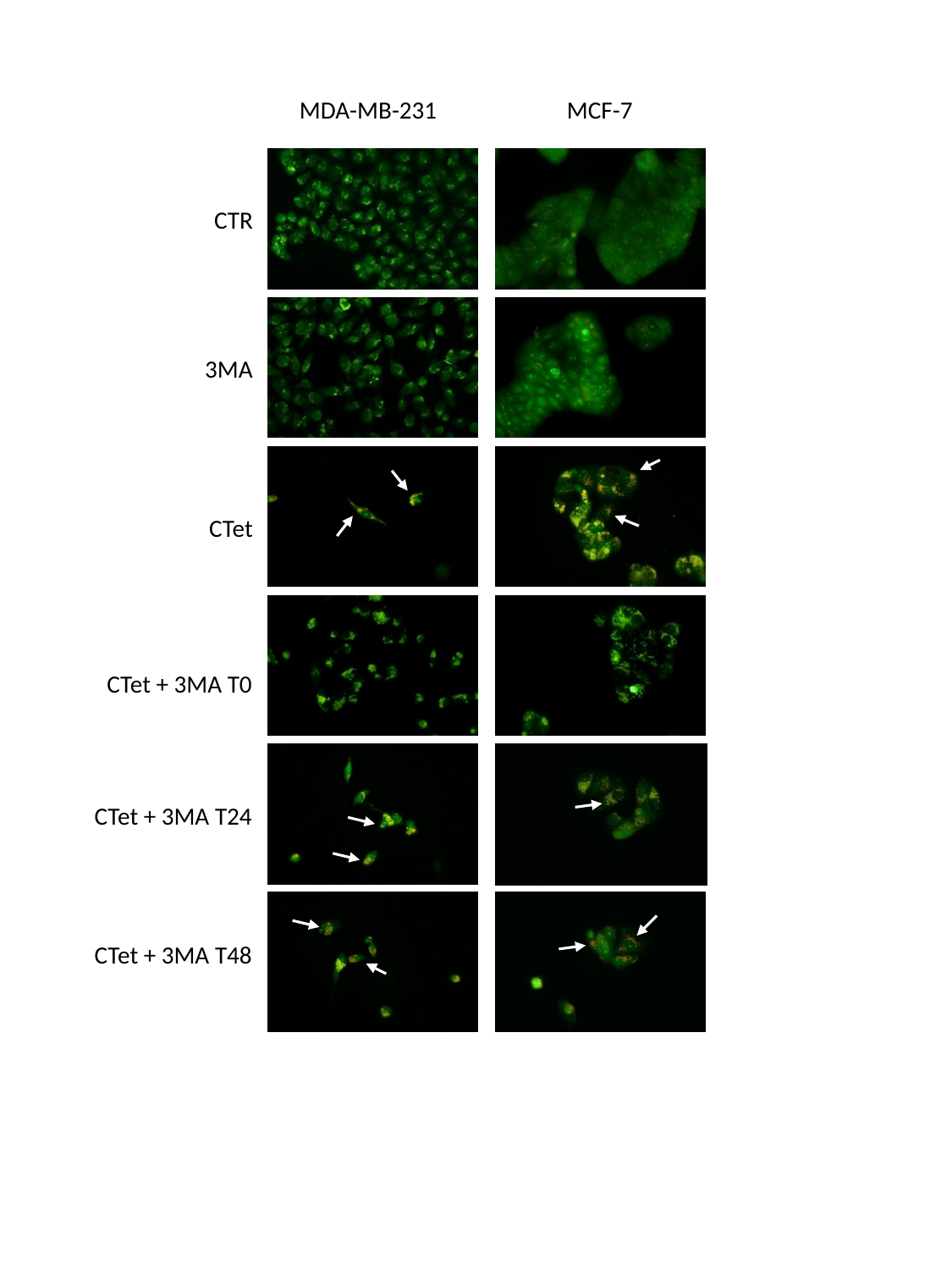

MDA-MB-231
MCF-7
CTR
3MA
CTet
CTet + 3MA T0
CTet + 3MA T24
CTet + 3MA T48

Supplement: Figure S2 — 3-MA effects on AVOs formation in MDA-MB-231 and MCF-7 cells. MDA-MB-231 (left panel) and MCF-7 (right panel) were treated with 8 µM CTet for 72 h and stained with acridine orange. 3-MA (1 mM) was added simultaneously with CTet (T0) or 24 h (T24) and 48 h (T48) after beginning CTet treatment to inhibit AVOs formation. Micrographs were taken using a fluorescent microscope (Blue excitation filter). The cytoplasm and nucleus of the stained cells fluoresced bright green, whereas AVOs fluoresced bright red. Results show that CTet induced AVOs formation in both cell lines, inhibited by 3-MA when added at T0. (PPT) [file pone.0043249.s002.ppt]

## Slide 1
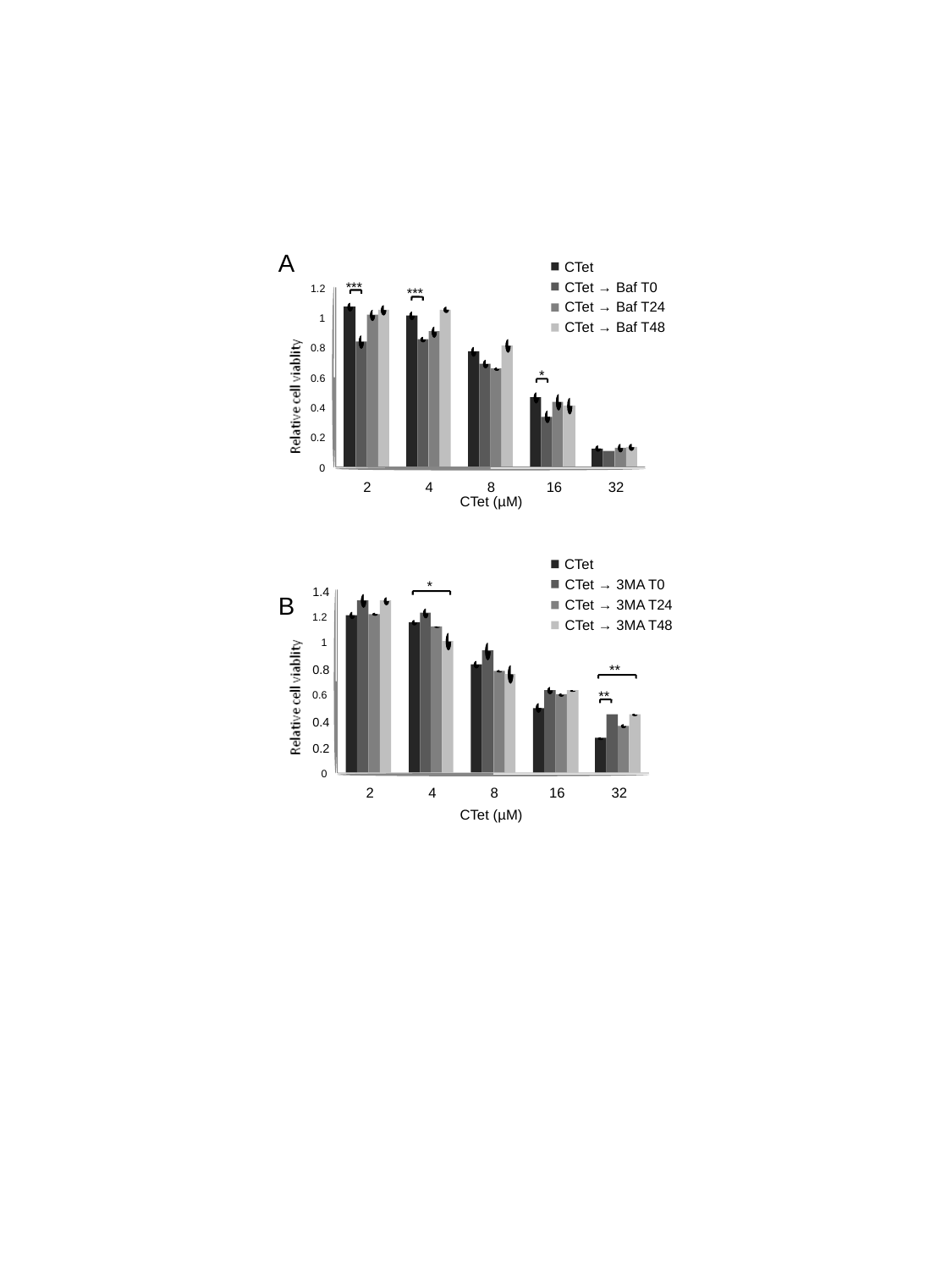

A
CTet
***
CTet → Baf T0
1.2
***
CTet → Baf T24
1
CTet → Baf T48
0.8
*
0.6
0.4
0.2
0
2
4
8
16
32
CTet (µM)
CTet
CTet → 3MA T0
*
1.4
B
CTet → 3MA T24
1.2
CTet → 3MA T48
1
**
0.8
**
0.6
0.4
0.2
0
2
4
8
16
32
CTet (µM)

Supplement: Figure S3 — Effect of autophagy inhibition in CTet-treated MCF-7 cells. MCF-7 cells were treated with increasing concentration of CTet and autophagy was pharmacologically inhibited at indicated time with 1 nM bafilomycin A1 (A) and 1 mM 3-MA (B). Inhibiting autophagy did not reduce CTet activity, except at the highest CTet dose, when autophagy inhibition occurred with 3-MA, indicating a minor role of autophagy in MCF-7 cell death. Data are expressed as relative cell viability normalized to Bafilomycin- and 3-MA-treated cells. Data are means ± SD of at least two experiments performed in triplicate. *p<0.05; **p<0.01; ***p<0.001. (PPT) [file pone.0043249.s003.ppt]

## Slide 1
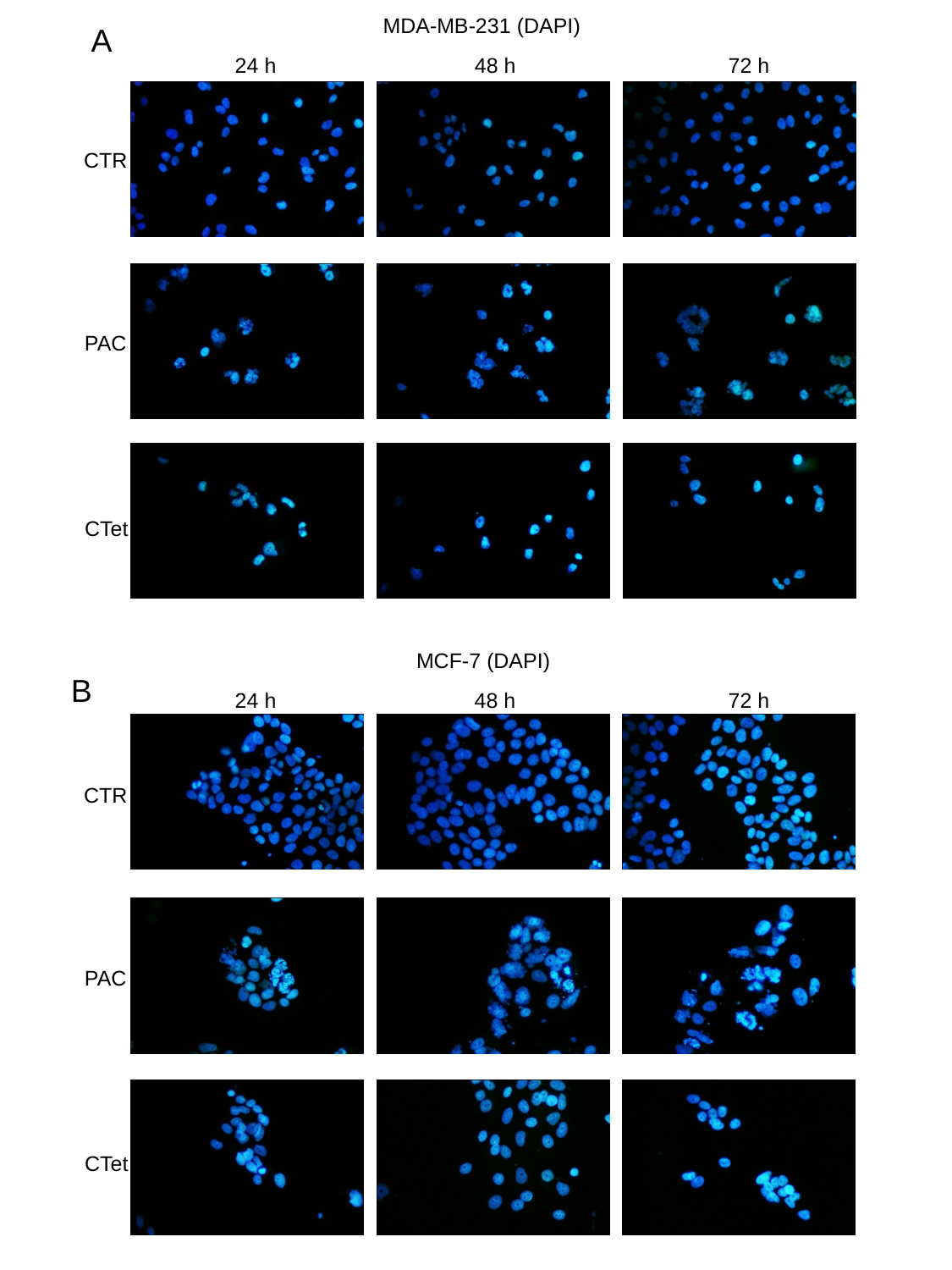

MDA-MB-231 (DAPI)
24 h
48 h
72 h
CTR
PAC
CTet
A
MCF-7 (DAPI)
24 h
48 h
72 h
CTR
PAC
CTet
B

Supplement: Figure S4 — Evaluation of apoptotic processes. MDA-MB-231 (upper panel) and MCF-7 (lower panel) were treated with 8 µM CTet for 24, 48 and 72 h and stained with DAPI for apoptosis evaluation. Paclitaxel was used as positive control. Results showed absence of apoptotic morphologic features (i.e. nuclear fragmentation) in both CTet-treated cell lines. CTR, control; PAC, Paclitaxel. (PPT) [file pone.0043249.s004.ppt]

## Slide 1
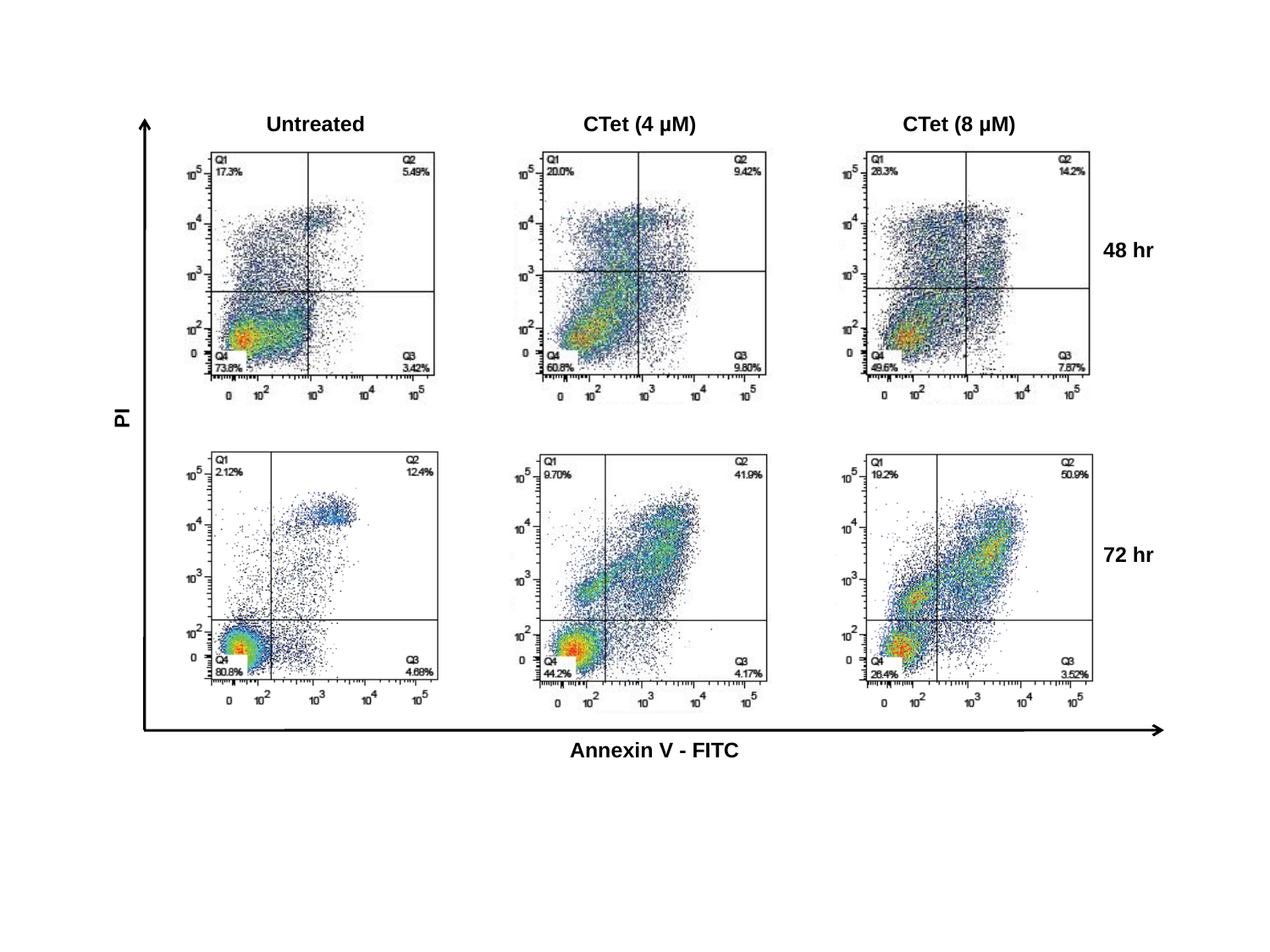

Untreated CTet (4 µM) CTet (8 µM)
48 hr
72 hr
PI
Annexin V - FITC

Supplement: Figure S5 — Evaluation of apoptosis/necrosis by Annexin V–PI staining. MDA-MB-231 cells were treated with 4 µM and 8 µM CTet for 48 and 72 h and double stained with Annexin V/PI. The amount of apoptotic (annexin V+/PI−) CTet-treated cells was always below 10%, while nonapoptotic CTet-treated cells (Annexin V+/PI+ plus Annexin V−/PI+) varied from 30% (4 µM CTet, 48 h treatment) to 70% (8 µM CTet, 72 h treatment). (PPT) [file pone.0043249.s005.ppt]

## Slide 1
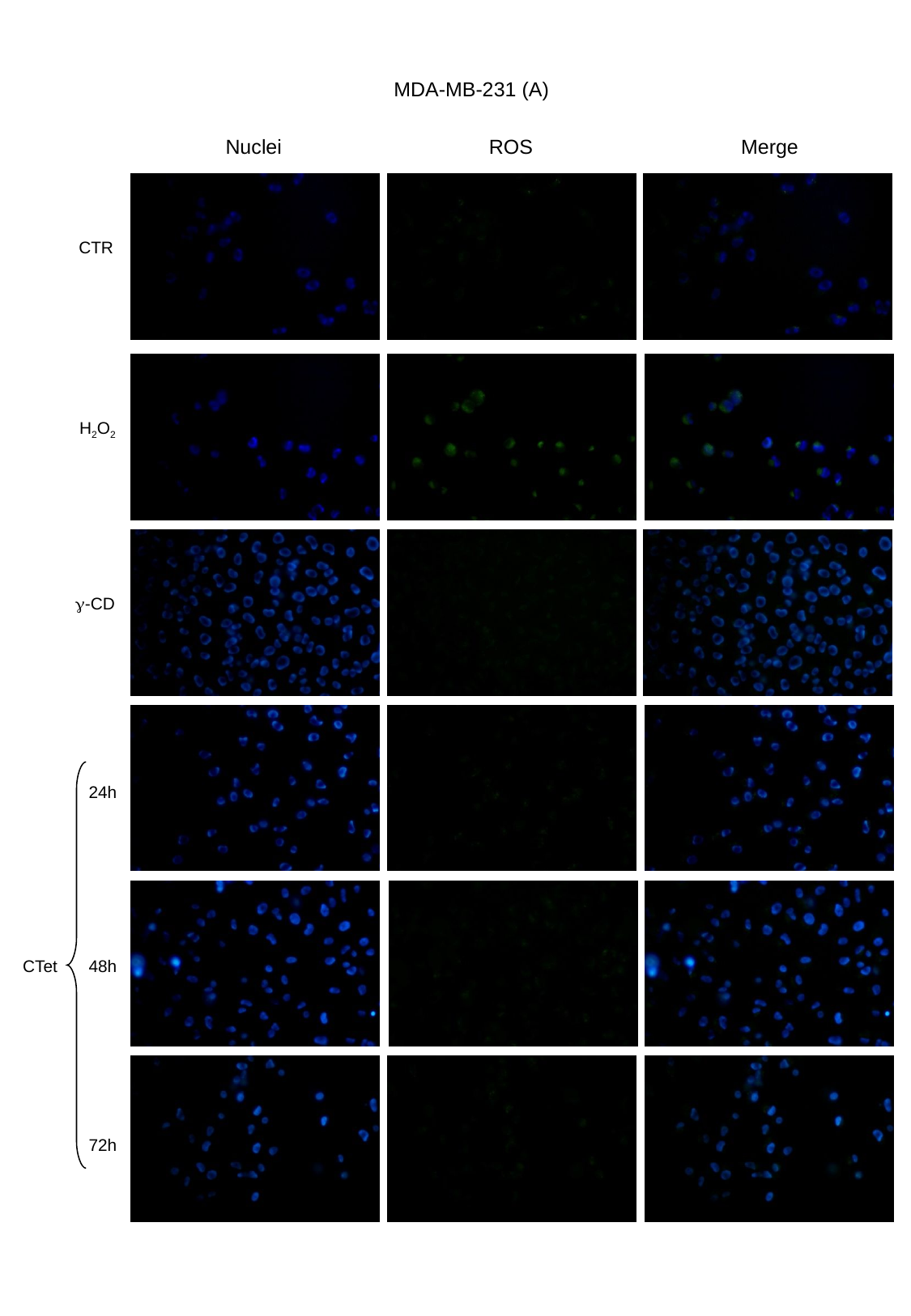

MDA-MB-231 (A)
Nuclei
ROS
Merge
CTR
H2O2
γ-CD
24h
CTet
48h
72h

## Slide 2
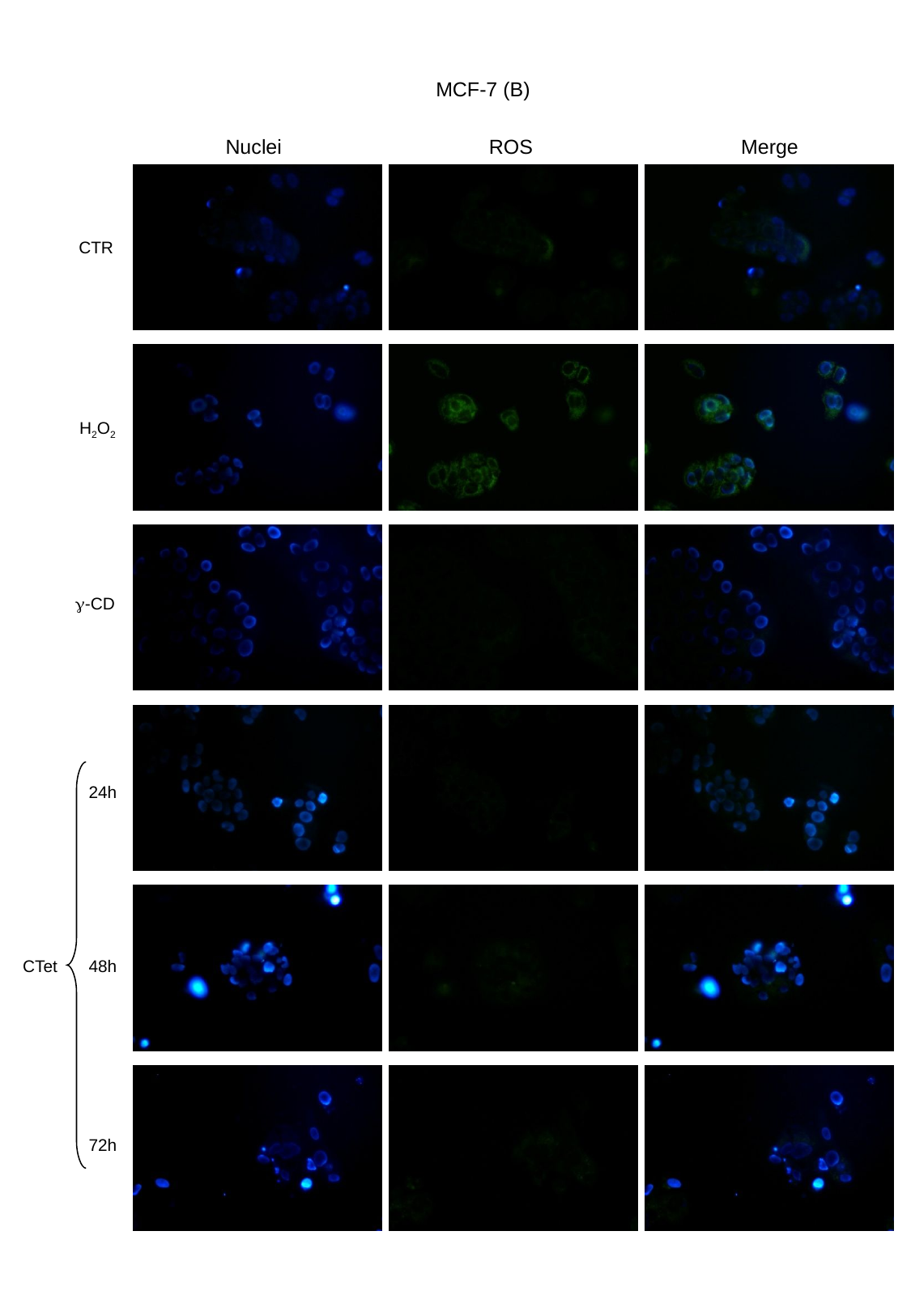

MCF-7 (B)
Nuclei
ROS
Merge
CTR
H2O2
γ-CD
24h
CTet
48h
72h

Supplement: Figure S6 — Detection of Reactive oxygen species (ROS). MDA-MB-231 (A) and MCF-7 (B) cells were treated with 8 µM CTet for 24, 48 and 72 h and incubated with DHR for 30 min. Nuclei were counterstained with Hoechst dye. Oxidized-DHR fluoresced bright green, whereas nuclei fluoresced blue. Results show that CTet did not induce ROS formation neither in MDA-MB-231 (A) nor in MCF-7 (B) cell lines. As positive control, cells were treated with 1 mM H2O2 for 1 h. CTR, untreated control; γ-CD, γ-cyclodextrin. (PPT) [file pone.0043249.s006.ppt]
